# A Framework for Rice Heavy Metal Stress Monitoring Based on Phenological Phase Space and Temporal Profile Analysis

**DOI:** 10.3390/ijerph16030350

**Published:** 2019-01-26

**Authors:** Xinyu Zou, Xiangnan Liu, Mengxue Liu, Meiling Liu, Biyao Zhang

**Affiliations:** School of Information Engineering, China University of Geosciences, Beijing 100083, China; zxy_cugb@163.com (X.Z.); mengxueliu25@163.com (M.L.); liuml@cugb.edu.cn (M.L.); zhangbycn@cugb.edu.cn (B.Z.)

**Keywords:** rice heavy metal stress, phenological phase space, remote sensing, spatiotemporal data fusion, time series

## Abstract

Previous studies make it possible to use remote sensing techniques to monitor heavy metal stress of rice synchronously and continuously. However, most studies mainly focus on the analysis of rice’s visual symptoms and physiological functions rather than temporal information during the growth period, which may reflect significant changes of rice under heavy metal stress. In this paper, an enhanced spatial and temporal adaptive reflectance fusion model was used to generate synthetic Landsat time series. A normalized difference water index and an enhanced vegetation index were employed to build phenological phase space. Then, the ratio of the rice growth rate fluctuation (GRFI Ratio) was constructed for discriminating the different heavy metal stress levels on rice. Results suggested that the trajectories of rice growth in phenological phase space can depict the similarities and differences of rice growth under different heavy metal stress levels. The most common phenological parameters in the phase space cannot accurately discriminate the heavy metal stress level. However, the GRFI Ratio that we proposed outperformed in discriminating different levels of heavy metal stress. This study suggests that this framework of detecting the heavy metal pollution in paddy filed based on phenological phase space and temporal profile analysis is promising.

## 1. Introduction

Heavy metal pollution is one of the most serious problems that influences the food security and public health at a regional and a global scale [1]. Traditional methods such as field investigation and laboratory analysis are widely used to acquire information of heavy metal pollution. These technologies of traditional methods are mature and have high measurement precision. However, they only obtain the pollution information around the sampling sites. Moreover, when it comes to large-scale agricultural pollution monitoring, it is laborious when using traditional methods. Therefore, there should be more efficient methods to meet the needs. Remote sensing has large-scale, real-time, and continuous characteristics in data acquisition, which makes it an important technology for monitoring heavy metal pollution in fields on a large scale [2,3,4].

Previous research has shown that the pigment content, dry matter content, photosynthesis, and transpiration of crops were changed under heavy metal stress [5,6,7]. These cause the changes in color, morphology, and physiological functions of crops as well as their spectral characteristics in remote sensing images [8,9,10]. Changes in spectral characteristics have become the basis of remote sensing monitoring of heavy metal stress in crops [11]. Currently, existing remote sensing methods that monitor the heavy metal stress can be categorized into three groups. The first is the spectral analysis of visual symptoms (color and morphology) in crops under heavy metal stress [11,12,13]. These methods could reflect the morphologic change under heavy metal stress and effectively evaluate the level of heavy metal stress in some ways. However, most of these relationship models of spectral characteristics under heavy metal stress were empirical models or semi-empirical models. Therefore, these methods cannot develop into a universal and mechanistic model to evaluate the heavy metal stress of crops. The second is the retrieval and analysis of physiological functions (chlorophyll content, cellular structure, photosynthesis, transpiration, and stomatal conductance) in crops under heavy metal stress [14,15,16,17]. Although these studies have achieved good results to assess the heavy metal stress level of crops using physiological functions, environmental factors may have a big impact on physiological functions, which causes difficulties in acquiring stable characteristics to assess the heavy metal stress level. The last is the crop growth model that is combined with remote sensing data assimilation to monitor heavy metal stress [18,19]. Nevertheless, the crop growth model is a kind of point model, and the accuracy of the model will be influenced by the spatial and temporal resolution of remote sensing data when remote sensing data assimilation comes to a regional scale. In addition, there are many processes that need further consideration when assimilation couples several complicated crop growth models.

All of the previously mentioned methods solve some problems in heavy metal assessment. However, they only take one mono-temporal image or several multi-temporal images for heavy metal stress monitoring of rice. They cannot reveal the continuously changing pace of the rice growth condition, and may neglect significant changes of the rice growth process under heavy metal stress. Therefore, sensitive and stable methods for the detection of heavy metal stress from information of the rice growth period are essential for agricultural, ecological, and food security. Crop phenology [20] is not only the sensitive indicator reflecting environmental changes, but also reveals the important information of ecosystem process. It provides important clues for studying the interaction between the environment and the ecosystem [21,22,23]. Therefore, monitoring the heavy metal stress of rice using phenological information from the time series data has been popular recently. Studies tried to use time series data [24] and vegetation phenological information [25] to assess the heavy metal stress level. They both used the greenness information (such as the normalized difference vegetation index, NDVI or the enhanced vegetation index, EVI) of rice. However, all aspects of rice besides greenness information are affected by heavy metal stress. Therefore, it is necessary to increase information to characterize other aspects of rice to jointly describe rice phenology and rice dynamics in order to enhance remote sensing signals of rice under heavy metal stress.

The fast development of spatiotemporal data fusion technology [26] and phase space theory provide new ideas for heavy metal stress monitoring. Spatiotemporal data fusion technology can be used to generate synthetic Landsat time series data, which can reflect the spatial and temporal information of landscapes. Phase space is an n-dimensional orthogonal data space that represents all possible states that the system possesses [27]. Behaviors of the system can be characterized by its motion and trajectory in the phase space. In remote sensing literature, feature space, band space, and spectral space are usually used to represent the concept of the phase space. The phase space of vegetation indices dynamics is a method that is used for characterization of phenology or the phenological phase space. It can overcome the uncertainties in phenology extraction and reflect the information in the vegetation growth process. In this paper, our focus was on extracting the stable and sensitive indicator for discriminating different stress levels of heavy metal from the whole rice growth period using the spatiotemporal data fusion method and the phenological phase space theory. This framework will be a promising approach for pollution investigation of the agricultural field and provides new ideas for monitoring the heavy metal stress of rice.

## 2. Materials 

### 2.1. Study Area

**Figure 1:**
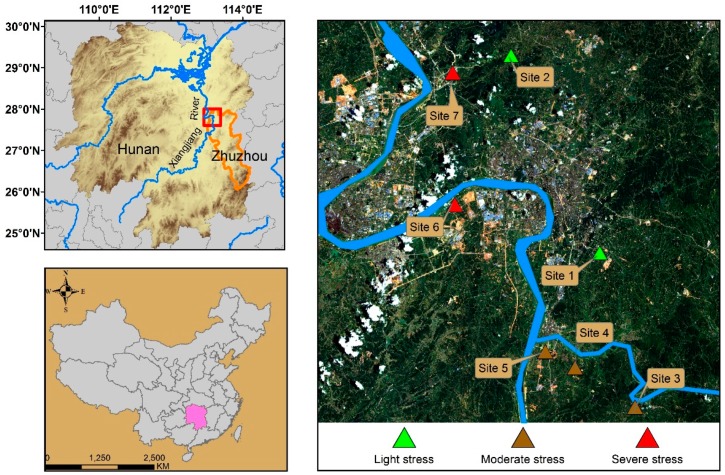
The location of the study area and the distribution of study sites (The green triangles represent the study sites under a light stress level of heavy metal, the dark brown triangles represent the study sites under a moderate stress level of heavy metal, and the red triangles represent the study sites under a severe stress level of heavy metal).

The study area is located in the Zhuzhou city along the Xiangjiang River Basin in the eastern part of the Hunan province (Figure 1). The geographical position is from 112.6° to 114° East longitude and from 26° to 28° North latitude. This area is in a subtropical monsoon climate, the annual average temperature is between 16 and 18 °C, and the soil type is mainly red soil, which is very suitable for planting rice. This is a famous grain yield area in the Hunan province, as well as an important commodity grain base in China. However, the Xiangjiang River Basin is a typical heavy metal pollution area in China. The Xiangjiang River and its tributaries have been polluted by industrial waste for a long time. The use of river water for irrigation in local farmlands has resulted in heavy metals pollution in farmland, which has caused crop growth to be stressed by heavy metals [28].

### 2.2. Field Sampling

Seven typical study sites in the study area were selected to collect field data of soil for measuring their heavy metal contents. Each study site is about 1.28 KM × 1.28 KM in size and contains about four to six paddy fields. Five soil samples and several paddy samples were randomly collected in each paddy field, and the weight of each soil sample was about 100 g. The geographical location of these samples was marked using a portable global positioning system (GPS). Lastly, the latitude and longitude of the samples at the center of the study site is selected as its geographical position, and the average heavy metal content of soil samples in each study site is taken as its heavy metal content. The main elements of heavy metal pollution in this study area are cadmium (Cd), lead (Pb), and mercury (Hg), among which Cd is the most significant. Therefore, we divide these seven sites into three categories based on the content of Cd, light stress, moderate stress, and severe stress. The heavy metal content of soil in each study site is shown in Table 1, and the overview of the study area and the distribution of the study sites are shown in Figure 1.

## 3. Method

**Figure 2:**
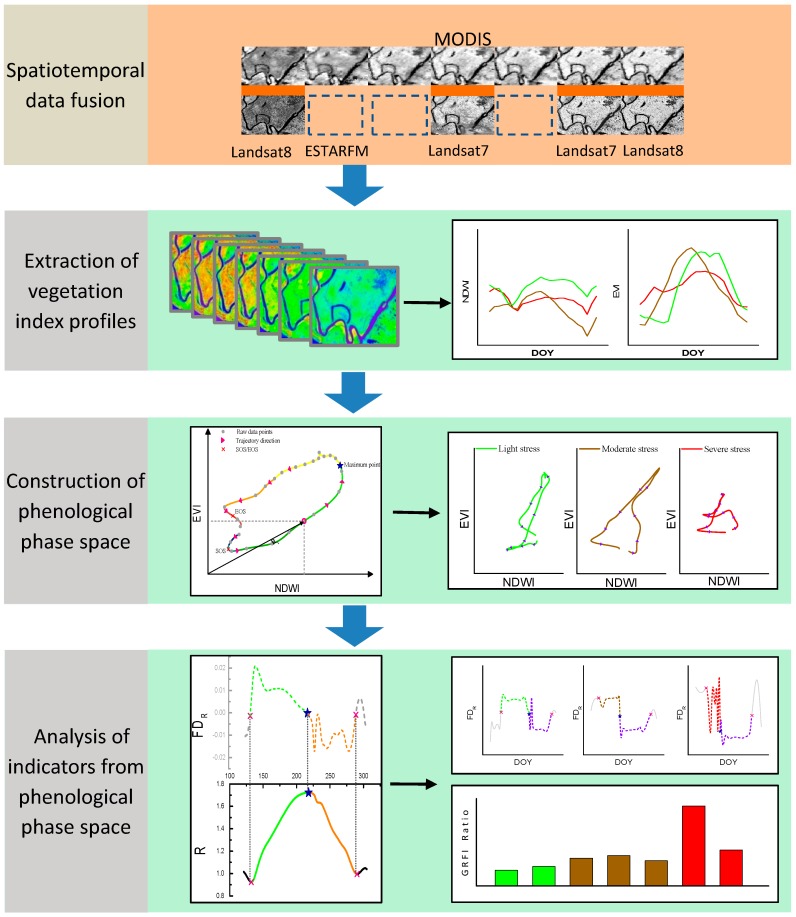
Flow chart of monitoring heavy metal stress of rice using phenological phase space and temporal profile analysis (ESTARFM represents enhanced spatial and temporal adaptive reflectance fusion model. NDWI represents the normalized difference water index. NDVI represents the normalized difference vegetation index. EVI represents the enhanced vegetation index. DOY represents the day of the year. R represents the distance from the data point of the *i*th day to the origin of the phase space. FD_R_ represents the first derivative of R. GRFI represents the rice growth rate fluctuation. The GRFI Ratio represents the ratio of the rice growth rate fluctuation.

In this paper, we generated synthetic Landsat time series of the study area by spatiotemporal data fusion. Then two different vegetation index temporal profiles, which could describe the process of rice growth in two different aspects were extracted from time series data. Thirdly, the phenological phase space was constructed. Lastly, we could analyze the differences in the process of rice growth under different heavy metal stress levels and constructed a stable and sensitive indicator for discriminating different heavy metal stress levels for the paddy field. The flow chart of monitoring heavy metal stress of rice using phenological phase space from synthetic remote sensing time series data is shown in Figure 2.

### 3.1. The Generation of Synthetic Landsat Time Series

#### 3.1.1. Data Preparation

Landsat-8 OLI, landat-7 ETM+, and MOD09A1 (MODIS surface reflectance products) are used for this study. All data available in the rice growing season between late April and late October in 2016 in the study area were obtained. The MOD09A1 product is eight-day composite surface reflectance data with a spatial resolution of 500 meters, while the Landsat series data have the spatial resolution of 30 meters with the temporal resolution of 16 days. The high-quality Landsat images (cloud cover less than 35%) in the study area were selected. There are seven images that are selected in total in which it contains four images from Landsat-8 OLI and three images from landsat-7 ETM+. The selected images from Landsat-7 ETM+ were restored by ENVI Landsat-gapfill part because of the problem of strips. The number of images cannot meet the need of constructing Landsat time series. Hence, we take the spatiotemporal data fusion algorithm to cover the shortage of data.

#### 3.1.2. Generation of Synthetic Landsat Time-Series Data using ESTARFM

**Figure 3:**
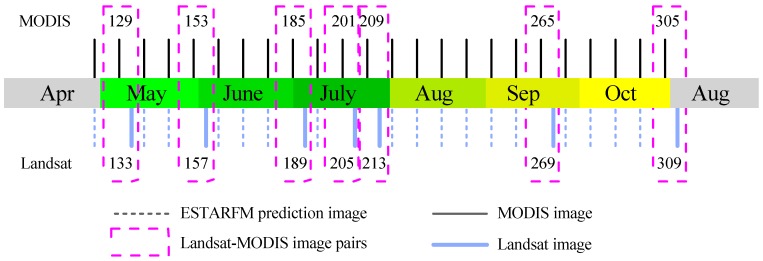
Date of MODIS, original Landsat images, and synthetic Landsat-like images by ESTARFM.

The spatio-temporal data fusion algorithm that we employed was the enhanced spatial and temporal adaptive reflectance fusion model (ESTARFM) [29]. This algorithm generates a fine-resolution image on the prediction date using two pairs of fine-resolution and coarse-resolution images acquired on the base date and a coarse-resolution image acquired on the prediction date. ESTARFM improves the accuracy of predicted fine-resolution image reflectance, especially for heterogeneous landscapes, and preserves spatial details [30]. Hence, it is very suitable to employ ESTARFM to blend MODIS and Landsat to generate time series data with eight-day temporal resolution and 30 meters of spatial resolution because of the fragment of landscapes in the study area. An overview of original Landsat data and synthetic images are displayed in Figure 3. To facilitate the narrative, we take the date as the day of the year (DOY).

The process of generating the high spatial and temporal resolution time series data contains four steps. First, we acquired the images of MODIS and Landsat that meet the quality requirements. Next, related data processes were conducted for preparation of data fusion, including radiance calibration, atmospheric correction, image clipping, and resampling. Third, the processed data were used as input data to carry out the ESTARFM, and the synthetic Landsat-like images were obtained. Lastly, we used the original Landsat images combining with the synthetic Landsat-like images to construct the time series data of eight-day temporal resolution and 30 meters of spatial resolution.

### 3.2. Extraction of Vegetation Index Profiles from Synthetic Landsat Time-Series Data

#### 3.2.1. Selection of Vegetation Indices

NDVI and EVI are two indices that were widely used in the study of vegetation phenology [31]. Meanwhile, the current extraction of vegetation phenology in remote sensing mostly depends on the characteristics of NDVI/EVI time series [15,32]. Yet, the greenness information cannot reveal some phenological characteristics, which do not take on a significant change in greenness under heavy metal stress. The normalized difference water index (NDWI) [33] is an index that measures the moisture content in vegetation canopy. Hence, it is feasible to apply NDWI to analyze rice canopy stress. EVI is an improved vegetation index, which directly adjusts the reflectance in the red band as a function of the reflectance in the blue band, and it accounts for residual atmospheric contamination and variable soil and canopy background reflectance [34]. Moreover, it is not easy to reach the saturation in regions covered with thick vegetation when compared to NDVI [35,36]. Therefore, we select EVI and NDWI to construct temporal profiles. They were calculated using the following equations.

(1) $$EVI=2.5\times \frac{\rho_{NIR}-\rho_{Red}}{\rho_{NIR}+6\times \rho_{Red}-7.5\times \rho_{Blue}+1},$$

(2) $$NDWI=\frac{\rho_{NIR}-\rho_{SWIR}}{\rho_{NIR}+\rho_{SWIR}},$$

where ρ_SWIR_, ρ_NIR_, ρ_Red_, and ρ_Blue_ stand for short wave infrared (SWIR) band, near infrared (NIR) band, red band, and blue band, respectively. Detailed information about these bands are shown in Table 2.

#### 3.2.2. Extraction of Vegetation Index Profiles

**Figure 4:**
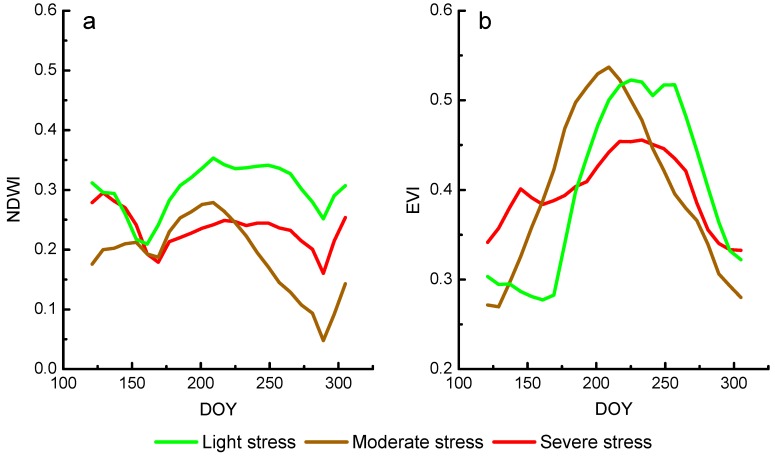
The NDWI (**a**) and EVI (**b**) temporal profiles after smoothing.

In this part, we extract EVI and NDWI temporal profiles of rice in study sites of three different heavy metal levels from time series data. The temporal profile indicates that the condition of vegetation growth should be continuous and smooth. However, the reflectance has been influenced by clouds, shadows, and aerosol in spite of strict pre-processing. It is necessary to smooth the temporal profile to reduce the impact of noise. In this study, we choose a widely used S-G filter to smooth the EVI and NDWI profiles to eliminate noise [37] and this smoothing process is carried out in TIMESAT software [38]. The smoothed average NDWI and EVI of study sites of different heavy metal stress levels are shown in Figure 4.

### 3.3. Construction of EVI-NDWI Phase Space

**Figure 5:**
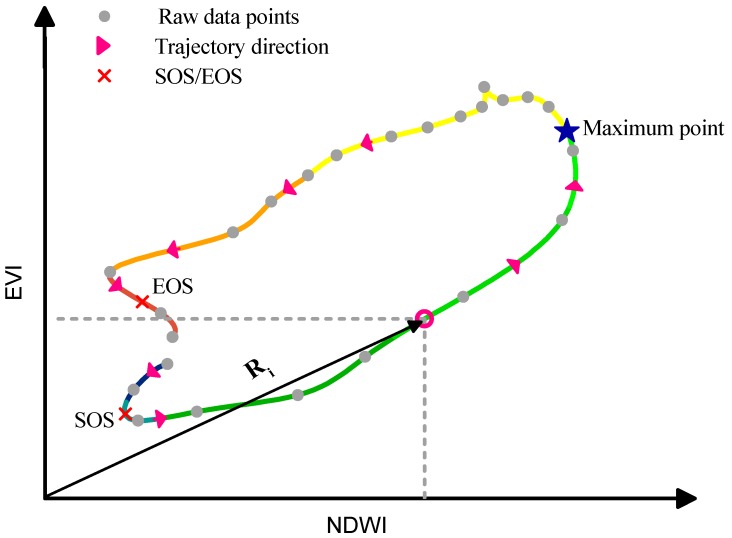
Rice phenological phase space and its phenological parameters, where SOS and EOS denote the beginning and the end of the rice growth period, respectively. *Ri* represents the distance from the data point of the *i*th day to the origin of the phase space and the maximum point is the data point that is farthest from the origin of the phase space.

In this study, the phenological phase space means that the data points in the rice growing period are marked in the phase space in order for the phase space to describe the rice growth process. The rice phenological phase space was constructed using EVI and NDWI in which NDWI is the x-axis and EVI is the y-axis. The data points marked in the phase space can be connected in a time sequence to form a profile. This profile represents the trajectory of rice growth. We can extract a series of phenological parameters to reveal the state of rice growth and even dig out the stress information it bore from the profiles. In order to make the profile smoother, it is formed by connecting the daily data generated by B-spline interpolation performed on these raw data points. The rice phenological phase space is shown in Figure 5. The start of the rice growing season (SOS), the end of the rice growing season (EOS), the maximum point, and *R_i_* in phase space were calculated as follows.

(3) $$SOS:\;Min\left(\sqrt{EVI_{i}^{2}+NDWI_{i}^{2}}\right),\;i\leq i\left(Max\right),$$

(4) $$EOS:Min\left(\sqrt{EVI_{i}^{2}+NDWI_{i}^{2}}\right),\;i\left(Max\right)\leq i,$$

(5) $$Maximum\;point:\;Max\left(\sqrt{EVI_{i}^{2}+NDWI_{i}^{2}}\right),$$

(6) $$R_{i}=\sqrt{EVI_{i}^{2}+NDWI_{i}^{2}},$$

### 3.4. Construction of Indicators of Phenological Phase Space

**Figure 6:**
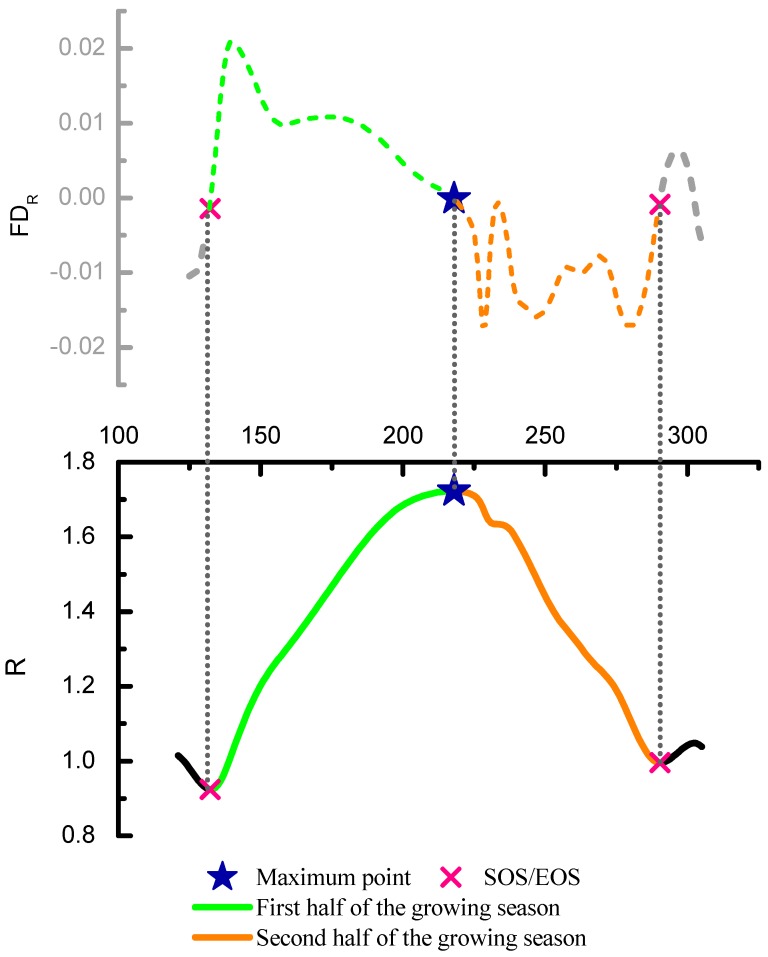
R and FD_R_ during the rice growing period. The maximum point divides the growth process of rice into two parts: the first half of the rice growth period and the second half of the rice growth period.

**Figure 7:**
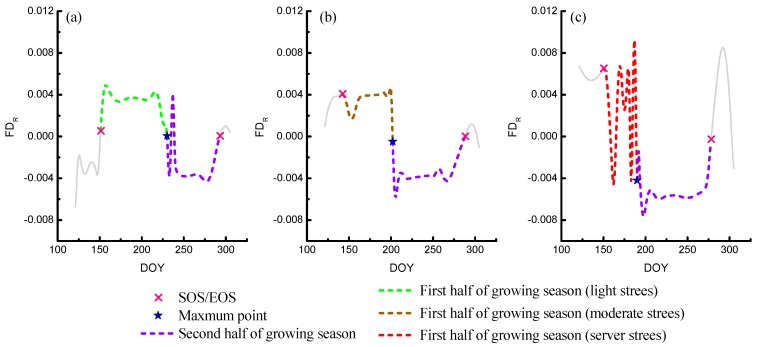
FD_R_ under different levels of heavy metal stress, including light stress (**a**), moderate stress (**b**), and severe stress (**c**).

The distance R in the phenological phase space can be considered as the characterization of the state of rice growth (Figure 6), and its first derivative (FD_R_) can represent the rice growth rate (Figure 7, Formula 7). During SOS to the maximum point of the rice growing season, if the value of FD_R_ changed slightly (Figure 7a), it could be considered that the growth of rice was relatively stable. If the value of FD_R_ changed drastically (Figure 7c), it could be considered that the growth of rice was fluctuating (unstable). In this paper, the rice growth rate fluctuation index (GRFI) was designed to measure the fluctuation of the rice growth rate, and it is obtained by calculating the standard deviation of the growth rate during a period of rice growth (Formula 8). The bigger the value of GRFI is, the more fluctuant (more unstable) the growth rate is, which indicates that the rice growth is affected by some disturbance. In these paddy fields with different heavy metal sampling areas, the intensive planting mode reduced the influence of soil nutrient content and spatial variability of soil texture characteristics. Furthermore, rice in these sites have been adequately cultivated and irrigated in the same climate conditions. Therefore, other environmental factors lead to the same stress on rice. Therefore, different heavy metal stress is the main disturbance factor causing the difference in rice growth within these study sites, which means that the different heavy metal stress can be discriminated by capturing these differences.

Furthermore, the ratio of the rice growth rate fluctuation (GRFI Ratio) correlated with heavy metal stress levels in rice, and it is obtained by dividing the value of GRFI in the first half of the rice-growing season in the second half of the rice growing season. Because previous research studies showed that the stress effects of heavy metals on rice in the first half of growth process is greater than that in the second half. Therefore, the more serious the heavy metal stress is, the great value the GRFI Ratio is. Therefore, the GRFI Ratio can indicate the level of heavy metal stress in rice. In addition, the GRFI Ratio is a kind of ratio-type indicator and can eliminate the interference of non-heavy metal stress factors between different sites to some extent. The rice growth rate (FD_R_), the rice growth rate fluctuation index (GRFI), and its GRFI Ratio formula are as follows.

(7) $${\mathrm{FD}}_{R}=\frac{\left(R_{\left(i+1\right)}-R_{i}\right)}{\Delta_{\mathrm{DOY}}},$$

(8) $$GRFI=\sqrt{\frac{1}{N}\overset{N}{\underset{i=1}{\sum }}{\left(FD_{R,i}-\bar{FD_{R}}\right)}^{2}},$$

(9) $$GRFI\;Ratio=\frac{GRFI{\;}_{SOS\leq i\leq Max}}{GRFI{\;}_{Max\leq i\leq EOS}},$$

## 4. Results

### 4.1. The Analysis of Rice Growth Trajectories under Different Heavy Metal Stress Levels

**Figure 8:**
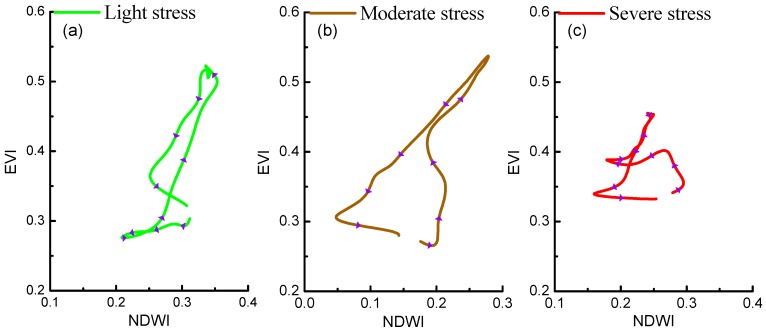
Rice phenological phase spaces of three different heavy metal stress levels (light stress (**a**), moderate stress (**b**), sever stress (**c**)). Violet arrows represent the trajectory direction.

Figure 8 shows rice phenological phase space in sites of three different heavy metal stress levels, which the values were obtained by averaging the value of rice pixels in corresponding study sites. The rice growth trajectories in Figure 8 reflect the difference of rice growth under different heavy metal stress levels more clearly than Figure 4.

During the whole growing season of rice under different heavy metal stress levels, its EVI and NDWI follow the general rule of rising first and then decreasing. It is clear that the directions of rice growth trajectories are counterclockwise, from below the phase space to the upper right of the phase space and back to the lower left of the phase space. However, rice growth trajectories under three different heavy metal stress levels still show differences in the phase space. The differences are mainly in three points: (1) Amplitude of EVI and NDWI of severe stress rice is smaller when comparing the moderate stress rice and the light stress rice, and the trajectory of severe stress rice covers the smallest area than others. (2) The maximum values of EVI and NDWI of severe stress rice are smaller than those of moderately stressed rice and light stress rice. (3) The direction of the severe stress rice growth trajectory changed more frequently than that of moderately stressed rice.

### 4.2. The Comparison of Phase Space Parameters under Different Heavy Metal Stress

The most common phenological parameters in the phase space were extracted, and the result is shown in Table 3. The length of the growth period is the date of the EOS to the SOS date. The maximum value of R is the maximum distance between the rice growth profile and the origin in the phase space. Amplitude of R is the difference between the maximum value of R and the minimum value of R. The length of rice growth trajectory is the length of rice growth trajectory from SOS to EOS. Generally, the length of growth period in site_6 and site_7 that bear the severe heavy metal stress is shorter than that of other sites. The maximum value of R in site_6 and site_7 is less than that of other sites as a whole. Meanwhile, both the values of amplitude of R and the length of rice growth trajectory in severe heavy metal stress sites are less than that of other sites. Although these general phenological parameters reflect the impact of different heavy metal stress on rice to some degree, we cannot accurately discriminate the heavy metal stress level using these parameters. It is essential to construct other sensitive parameters in phase space to indicate the heavy metal stress.

### 4.3. The Comparison of GRFI Ratio under Different Heavy Metal Stress

**Figure 9:**
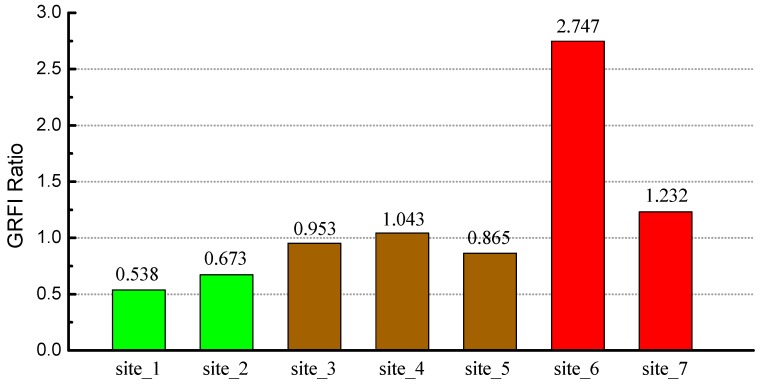
The ratio of rice growth rate fluctuation (GRFI Ratio) in different study sites.

Figure 9 shows the GRFI Ratio in study sites of different heavy metal stress levels. It is clear that there is an obvious difference in the GRFI Ratio of rice under different heavy metal stress levels. The values of the GRFI Ratio under light stress were relatively small, and the values of that in three sites under moderate stress were at an intermediate level, while those under severe stress had the largest values. Among them, the maximum GRFI Ratio appeared at site 6 of severe stress level, and the minimum GRFI Ratio appeared at site 1 of the light stress level. The smallest value of the GRFI Ratio in sites of severe stress is larger than the largest value of that in sites of moderate stress. The rule can also be found in sites of moderate stress and light stress. The results showed that there had been an obvious correlation between the GRFI Ratio and the heavy metal stress level. The more severe heavy metal stress level of rice is, the greater the value of the GRFI Ratio is.

Data in Figure 9 suggest that: (1) The values of the GRFI Ratio in the sites with a light stress level is small, and the difference of the GRFI Ratio between the light stress and the moderate stress level is also small. It can be considered that the two stress levels have little effect on rice growth and the difference is not clear. (2) The difference of the GRFI Ratio between sites with moderate stress is less clear than the difference of the GRFI Ratio between sites with other stress levels, which indicates that moderate stress levels have a significant and stable effect on rice growth. (3) There is a large difference of the GRFI Ratio between the two sites with a severe stress level. It shows that severe stress levels have serious effects on rice growth or may even cause disastrous consequences.

## 5. Discussion

The experimental results demonstrated that the rice growth trajectories under different heavy metal stress levels showed a difference in the phenological phase space, and the GRFI Ratio we proposed performed better in discriminating different heavy metal levels than the most common phenological parameters in the phase space. The paper has two innovations. One is the introduction of temporal information of rice growth period in two different aspects to monitor the heavy metal stress level of rice. Former studies [39,40] ignored the temporal information of the growth period of rice, which may reflect significant changes of rice [24] under heavy metal stress. Although some studies [25,41] have been conducted to monitor heavy metal stress of rice using NDVI/EVI temporal profiles from time series, the greenness information of rice may not fully reflect the influence of rice under heavy metal stress [42]. Hence, the NDWI combined with EVI was taken to show different aspects of rice under heavy metal stress. Another is the construction of a stable and sensitive indicator from the framework based on phenological phase space and temporal profile analysis for rice heavy metal stress monitoring. Results show that the GRFI Ratio that we constructed was an effective and stable indicator to discriminate heavy metal stress levels compared to other general phenological phase space parameters.

However, some limitations also exist in this study. First, the study sites are all located in the Zhuzhou city, and only remote sensing data of 2016 were employed to conduct the temporal profile analysis because of the limitation of field validation. The introduction of the study area in different climatic conditions and remote sensing data of more years will be taken into consideration in our further work. Moreover, the values of EVI/NDWI were obtained by averaging the values of rice pixels, which would lead to the problem of mixed pixels [43]. Better methods and techniques are needed to solve this problem. Subsequent studies could calculate the value of EVI/NDWI pixel by pixel if they can solve the problem of workload. In addition, due to the tradeoff of satellite, we cannot acquire both high spatial and temporal resolution data. The time series data were produced by the spatiotemporal data fusion method. However, synthetic data by the spatiotemporal data fusion method cannot replace the original images because of various errors [44,45]. These errors have an impact on the generation of time series data [46,47]. Therefore, remote sensing data with high spatial and temporal resolution are promising for obtaining the generation of time series data in the future with the development of science and technology. Lastly, this paper constructed two-dimensional phenological phase space to conduct the study, but the phase space are not limited to two-dimensional spaces. The n-dimensional phase space may reflect notable differences with two-dimensional phase space because it contains more information than the two-dimensional phase space.

## 6. Conclusions

In this paper, we focus on constructing a stable and sensitive indicator to monitor heavy metal stress of rice using phenological phase space theory combining with spatiotemporal data fusion technology. We first built the time series data of eight-day temporal resolution and 30 meters spatial resolution using the spatiotemporal data fusion algorithm. Then, we extracted the EVI and NDWI temporal profiles to construct the phenological phase space and plotted the growth trajectories of rice in the phase space. Lastly, a sensitive indicator GRFI Ratio for heavy metal stress in rice was constructed and results showed it outperformed in discriminating different heavy metal stress levels. The most important findings and conclusions drawn from this study include:

(1) The framework of rice heavy metal stress remote sensing monitoring consisting of the generation of synthetic Landsat time-series data and the construction of phenological phase space is feasible.
(2) The phenological phase space and the rice growth profiles can well depict the growth process of rice, and also reflect the effects of different heavy metal stress levels for rice growth.
(3) The GRFI Ratio was the stable and sensitive indicator, which we constructed from phenological phase space for discriminating heavy metal stress for rice. Comparing with other common phenological parameters in the phase space, the GRFI Ratio performed better.

In summary, a spatiotemporal data fusion method combining with phenological phase space technique can provide a stable and accurate method for detecting a heavy metal stress level in the paddy field. These technologies can be applied in pollution investigation of agricultural field, and provide new ideas for monitoring heavy metal stress.

## Figures and Tables

**Table 1:** Heavy metal content of soil samples in seven sites of study area (mg·kg^-1^).

Study site	Longitude	Latitude	Cd	Pb	Hg	Pollution Level
Site_1	27°47′ N	113°10′ E	0.84	78.33	0.35	Light stress
Site_2	27°59′ N	113°06′ E	1.38	59.45	0.35	Light stress
Site_3	27°37′ N	113°14′ E	2.25	89.67	0.29	Moderate stress
Site_4	27°40′ N	113°10′ E	2.31	91.05	0.24	Moderate stress
Site_5	27°41′ N	113°08′ E	2.71	87.2	0.29	Moderate stress
Site_6	27°50′ N	113°02′ E	3.28	120.75	0.51	Severe stress
Site_7	27°58′ N	113°02′ E	3.57	130	0.503	Severe stress
Background value			1.43	82.78	0.2	
National secondary standard			0.3–1.0	250–350	0.3–1.0	

Note: The concentrations of heavy metals were measured in dry weight, and the unit of heavy metal content is mg·kg^-1^. Background values of heavy metals were derived from the Hunan Institute of Geophysical and Geochemical Exploration, China. Cadmium content between the national secondary standard and the background value is defined as a "light stress" level, more than twice the background value is defined as a "severe stress" level, and greater than the background value but less than twice the background value is defined as a "moderate stress" level.

**Table 2:** Band information of MODIS, Landsat-8 OLI, and Landsat-7 ETM+.

Band	MODIS (MOD09A1)		Landsat-8 OLI		Landsat-7 ETM+	
	Band Number	Wavelength Coverage (μm)	Band Number	Wavelength Coverage (μm)	Band Number	Wavelength Coverage (μm)
Blue	Band 3	0.459–0.479	Band 2	0.452–0.512	Band 1	0.450–0.515
Green	Band 4	0.545–0.565	Band 3	0.530–0.590	Band 2	0.525–0.605
Red	Band 1	0.620–0.670	Band 4	0.636–0.673	Band 3	0.630–0.690
NIR	Band 2	0.841–0.876	Band 5	0.851–0.879	Band 4	0.775–0.900
SWIR	Band 6	1.628–1.652	Band 6	1.566–1.651	Band 5	1.550–1.750

**Table 3:** The general rice phenological parameters in the phase space.

	Site_1	Site_2	Site_3	Site_4	Site_5	Site_6	Site_7
Cadmium content in soil (mg/kg)	0.84	1.38	2.25	2.31	2.71	3.28	3.57
Length of growth period	142	127	151	148	146	90	97
Maximum value of R	0.621	0.691	0.741	0.675	0.605	0.635	0.518
Amplitude of R	0.275	0.296	0.352	0.301	0.256	0.158	0.116
Length of rice growth trajectory	0.552	0.762	0.815	0.673	0.605	0.380	0.279

Note: R represents the distance from the data point of the *i*th day to the origin of the phase space in Figure 5.
